# Letter from E. K. Hutchins

**Published:** 1868-09-15

**Authors:** E. K. Hutchins

**Affiliations:** Philadelphia


### *PHILADELPHIA CORRESPONDENCE.*

PHILADELPHIA, *Sept.* 18, 1868.

*Editor* CHICAGO MEDICAL JOURNAL:

The tendency towards *speciality* in our profession is daily increasing. Young men, of enlarged views and intelligent minds, are graduated from our colleges, and enter upon a *special* field of work at once. Notwithstanding the ridicule that has been thrown out, or *attempted* to be thrown out, against this class of onr profession, it is, nevertheless, an indisputable and recognized fact that the idea is becoming a fashionable one.

Fashion is just as much a tyrant in medicine as it is in the tailor establishment of our modern dandy, or the elegant dress emporium of our *elite* females. And this is not new or utopian. Several years ago, the late Professor Green, of New York, astounded the world by his proclamation of his treatment for throat and even chest affections. Immediately throat affections became *fashionable*, and thousands of mouths were opened to have the *swab* pushed down the throat. Louis XIV. was a sufferer with anal fistula, and in France this disease became at once so fashionable that surgeons were busy with cutting for this alone; "those who had only a *small draining* run immediately, and *turned up their posteriors.*" In 1840 and '41, when Dupuytren's operation for relaxation of the sphincter ani was in vogue, it became popular in this country, especially in New York, where *every thing* is in excess, and it is said the result was that "New York anuses looked like *gimlet holes in a piece of pork.*" And fashion *still* holds a powerful sway in our profession, but whilst we are embracing specialities more largely than heretofore, we are doing so with more discretion and intelligence. There is, too, that cordial and genial feeling entertained by the profession, as a class, towards specialists which is so impressively becoming to the fraternity. And this feeling is growing warmer and more extended every day. My object in these few prefatory remarks is to bring into notice that speciality which is becoming so popular among us now, and which, perhaps, of all others, is the object of the most uncharitable remark. I mean that of uterine diseases. Dr. Buck, of New Hampshire, two years ago, in an address before the medical society of that State, devotes about two pages in the printed matter, to the ridicule of these diseases. My purpose is to occupy a few pages of your journal in a series of two or three letters, illustrating, by cases, accurately and concisely given, the great prevalence of uterine diseases, and the value and importance of devoting *special* attention to them. To show, if possible, that the "*raid* being made upon the uterus at this time" is in the hands of intelligence, the means of averting much distress, and relieving much suffering, and to show that this "harmless, *inoffensive* little organ, stowed away in a quiet place," when *roused*, is the means of wrecking life, making pleasure a burden, and arresting, to an alarming extent, the furtherance of the race. And if proper treatment in this direction is observed, and these results *can* occur, it is *good* that there are those who "mount a speculum."

In my next letter I shall notice uterine disease *in general*, its unprecedented prevalence, its causes and its tendency. In letters No. 3 and 4 some notes will be made of special uterine diseases, together with some remarks on their treatment. As far as I can, I shall illustrate my subject with interesting cases.

E. K. Hutchins.

